# Indole modulates cooperative protein–protein interactions in the flagellar motor

**DOI:** 10.1093/pnasnexus/pgac035

**Published:** 2022-05-13

**Authors:** Rachit Gupta, Kathy Y. Rhee, Sarah D. Beagle, Ravi Chawla, Nicolas Perdomo, Steve W. Lockless, Pushkar P. Lele

**Affiliations:** a Artie McFerrin Department of Chemical Engineering, Texas A&M University, College Station, TX 77843-3122, USA; b Department of Biology, Washington University in St. Louis, St. Louis, Missouri, 63130, USA; c Department of Integrative Structural and Computational Biology, Scripps Research, La Jolla, CA 92037, USA; d Department of Biology, Texas A&M University, College Station, TX 77843-3258, USA

**Keywords:** exometabolome, PMF, chemotaxis, switching, chemoreceptors

## Abstract

Indole is a major component of the bacterial exometabolome, and the mechanisms for its wide-ranging effects on bacterial physiology are biomedically significant, although they remain poorly understood. Here, we determined how indole modulates the functions of a widely conserved motility apparatus, the bacterial flagellum. Our experiments in *Escherichia coli* revealed that indole influences the rotation rates and reversals in the flagellum’s direction of rotation via multiple mechanisms. At concentrations higher than 1 mM, indole decreased the membrane potential to dissipate the power available for the rotation of the motor that operates the flagellum. Below 1 mM, indole did not dissipate the membrane potential. Instead, experiments and modeling indicated that indole weakens cooperative protein interactions within the flagellar complexes to inhibit motility. The metabolite also induced reversals in the rotational direction of the motor to promote a weak chemotactic response, even when the chemotaxis response regulator, CheY, was lacking. Experiments further revealed that indole does not require the transporter Mtr to cross the membrane and influence motor functions. Based on these findings, we propose that indole modulates intra- and inter-protein interactions in the cell to influence several physiological functions.

## Introduction

Microbial niches are characterized by numerous biological molecules and metabolites—together termed as the exometabolome. Compounds in the exometabolome can be powerful regulators of the physiology of bacteria both native and foreign to the niche (1-3). A key component of the exometabolome in the gastrointestinal tract is indole, which is produced in copious amounts from tryptophan by several bacterial species (4-6). Indole regulates diverse cell functions including division, energetics, biofilm formation, and antibiotic resistance (4, 6-8). Indole is multifaceted—it influences numerous aspects of bacterial–mammalian interactions (9, 10), cell signaling (6, 8), inflammation (9), hormonal secretion (11), and plant defenses (12). However, a general understanding of its wide-ranging effects on physiological functions is lacking.

The membrane potential along with the transmembrane pH gradient—together termed as the proton-motive force (PMF)—powers motility in *Escherichia coli*. As indole dissipates the membrane potential at high concentrations (>3mM) (13), motility is expected to be sensitive to indole’s ionophoretic activity. Motility is enabled by a transmembrane flagellar motor that rotates an extracellular flagellar filament to generate hydrodynamic thrust on the cell (14). The motor switches between clockwise (CW) and counterclockwise (CCW) rotation to help the cell to swim toward favorable chemical habitats—a process termed as chemotaxis (15). Previously, we reported that indole is simultaneously sensed by two major chemoreceptors—Tar and Tsr—leading to a biphasic chemotaxis response. The complex chemotaxis response to indole promoted selective colonization of indole-rich niches by some cells, while others were repelled (16). Thus, indole is capable of influencing the composition of bacterial communities by modulating chemotaxis behavior.

The flagellar motor consists of a multimeric switch complex that lies at the base of the motor. The chemotaxis response regulator, CheY-P, binds to the switch to *allosterically* control its overall conformation, thereby promoting CW rotation in an otherwise CCW-rotating motor in *E. coli* (17, 18). Chemoreceptors modulate CheY-P levels to control flagellar switching in response to the detection of extracellular ligands. However, we previously found that indole could modulate flagellar switching independent of the chemoreceptors (16). Although the mechanisms of this receptor-independent modulation of switching are unknown, they are of significant interest as they could help indole elicit chemotaxis even in those bacterial species that lack indole-specific chemoreceptors. The manner of indole’s entry into the cell is debated—one group suggested that indole does not require transporter proteins to freely permeate liposome; however, others have implicated the permease Mtr in the transport of exogenous indole into bacterial cells (7, 19, 20). It is possible that indole enters into the cytoplasm and interacts with the motor complexes to influence switching.

Here, we dissected the effects of indole on motility in *E. coli.* Our experiments and stochastic modeling results were consistent with the notion that indole enters the cytoplasm and weakens cooperative interactions in the flagellar complexes to inhibit motility. Indole also decreases the free energy differences between the tensed and the relaxed conformations of flagellar proteins to elicit receptor-independent chemotaxis responses. Our measurements further indicate that the permease Mtr is not necessary for indole transport across the membrane.

## Materials and Methods

### Strains

All the strains were derived from *E. coli* RP437 (Table 1). For the motility assays, we employed strains carrying the native *fliC* allele on the chromosome. For the tethered cell and bead assays, we worked with strains that carried a sticky *fliC* variant in place of the chromosomal *fliC* allele. We used a one-step inactivation with the λ-Red-mediated homologous recombination technique to prepare the *mtr* mutant. The DNA for transformation was PCR-amplified from the Keio collection strain (#JW3130). The insertion was sequenced with a set of primers that complemented the flanking regions of the *mtr* gene plus the kanamycin resistance marker.

### Cell culture

We streaked fresh colonies on LB plates from frozen stocks for each experiment. A single colony was picked to start an overnight culture in 5 mL TB (tryptone broth) at 30°C. The following day, we inoculated 100 μL of the overnight culture in 10 mL fresh TB in a shaker incubator to grow a day culture to an OD600 = 0.5 at 33°C.

### Motility and bead assays

Cells were washed 2x (1,500 *g,* 5 min) via centrifugation in motility buffer (MB)—10 mM potassium phosphate buffer, 67 mM NaCl, 0.1 mM EDTA, 1 μM methionine, 10 mM sodium lactate, and pH 7.0. All buffer solutions were supplemented with 0.2–0.8% Dimethyl sulfoxide (DMSO) to facilitate the dissolution of indole; DMSO was added to the buffer for control experiments as well. For motility assays, the cells were imaged under the microscope. For bead assays, the cell suspension was first sheared by forcing it repeatedly through a 10 cm long polyethylene tubing (0.58 mm inner diameter). The cells were then adhered to a 12 mm diameter coverslip and the beads were attached to the sheared flagellar stubs. To adhere the cells, the coverslip was treated with 0.01% Poly-L-lysine for 5 min followed by a wash in copious amount of deionized (DI) water. The coverslip was mounted in a perfusion chamber that was connected via a 3-way pneumatic valve (Hamilton Inc.) to reservoirs of different buffer solutions. We continuously exchanged the liquid medium in the perfusion chamber by withdrawing the fluid with a syringe pump (Fusion 200, Chemyx) at a flow rate of 200 μL–250 μL/min. The valve enabled us to switch the reservoir source as desired to introduce different effectors in the chambers. We imaged the cells on a Nikon microscope (Optiphot 2) with a 10x/20x phase objective at 80 fps or 500 fps using a digital camera (UI-3240LE-M-GL or Fastech Imaging IL5LM8-1TB). We quantitatively analyzed videos of swimming cells and the rotation of beads using custom-written MATLAB scripts based on particle-tracking algorithms (24, 21).

### Fluorescence measurements

Following the 2x wash in MB, we washed the cells again in an MB solution containing the Thioflavin T (ThT) dye (10 *μ*M). We resuspended the cell pellet in the same MB-ThT solution. All the reservoirs (MB-only and MB-indole solutions) contained 10 *μ*M ThT. Once the cells were adhered to the coverslip and loaded into the perfusion chamber, we continuously perfused the MB-ThT solution into the chamber. During the experiment, we excited the cells with an LED light (SOLA SE Light Engine, Lumencor, Inc.) filtered with a 435/20 nm excitation filter (Nikon Inc.) on Ti-E Nikon microscope. The emissions were collected with a water immersion objective (60X, NA 1.2, Nikon Inc.) and passed through a 525/50 nm emission filter (AVR optics). The signal was relayed to a sensitive photomultiplier (H7421-40 SEL Hamamatsu Corp.) and sampled at 10 Hz (25). Since the wash in MB-ThT solution, the cells had ~30 min to equilibrate with the dye prior to imaging. The excitation intensity, focus, and cell coverage were maintained the same between all biological replicates.

### Calculations of relative changes in membrane potentials

We assumed that the mean prestimulus emission intensity (I_pre_) was proportional to the basal membrane potential in the cell population. To account for the differences in the prestimulus intensity due to varying cell coverage between the different treatments, we normalized the time-dependent signal in each experiment with its Ipre, which yielded the basal intensity per cell. We calculated the fractional decrease in the emission intensity per cell upon treatment with a ligand as

$$\Delta F_{\text{indole}}=(I_{\text{indole}}-I_{\text{pre}})∕I_{\text{pre}}.$$

Here, I_indole_ indicates the steady-state poststimulus intensity. Next, we separately quantified the maximum possible change in the membrane potential. The maximum change was obtained from the fractional decrease in the signal, ΔF_CCCP_, when the cell population was treated with 25 *μ*M Carbonyl cyanide m-chlorophenyl hydrazone (CCCP) as this concentration of CCCP completely dissipates the membrane potential (26):

$$\Delta F_{\text{CCCP}}=(I_{\text{CCCP}}-I_{\text{pre}})∕I_{\text{pre}}.$$

We calculated the relative change in the membrane potential as

$$\Psi_{F}=100\times (\Delta F_{\text{CCCP}}-\Delta F_{\text{indole}})∕\Delta F_{\text{CCCP}}.$$

A Ψ_F_ = 100% indicated no dissipation in the membrane potential, whereas Ψ_F_ = 0% indicated complete dissipation.

### Measurement of oxygen consumption rates

The rate of oxygen consumption was measured using a high-resolution O2K-FluorRespirometer (Oroboros), with a chamber temperature of 25°C, chamber volume of 2 mL, and a stirrer speed of 350 rpm. Overnight cultures were grown in TB (30°C, 250 rpm aeration) and diluted 1:100 into 5 mL of prewarmed TB. Day cultures were incubated at 33°C, 170 rpm aeration until the midexponential phase (OD_600_ ~ = 0.5). Cells were pelleted via centrifugation at 4,000 rpm for 3 min, and the pellets were resuspended in prewarmed MB to an OD_600_ = 0.5. A Hamilton syringe was used to inject 100 *μ*L of resuspended cells into chambers containing either MB + DMSO or MB + DMSO + 2 mM indole. The oxygen consumption rate was monitored for 15 min and was normalized to cell number.

### ATP measurements

The relative ATP levels were measured using Spectra Max Gemini EM ROM v2 with Promega’s BacTiter-Glo Microbial Cell Viability kit in 96-well half-area flat bottom plates (opaque). Luminescence was measured at emission wavelength of 542 nm. We followed the vendor’s protocol in preparing a standardized ATP curve to evaluate the working range of the instrument and to measure the relative ATP levels of the cell culture. We found that for our setup, luminescence depended linearly on ATP level between 10 and 1,000 nM, corresponding to 100–10,000 relative Luminescence unit. We prepared standardized dilutions of the cell culture to contain a total ATP between 10 and 1,000 nM. Then, cells were loaded on to the 96-well plate followed by addition of 2 mM indole to well. The mixture was gently mixed and incubated for 5 min, followed by mixing and incubation with BactTiter-Glo reagents before measurements.

### Statistical analysis

Student’s t test was used for all statistical analysis. Results with *P*-value <0.05 were considered statistically significant. Sample sizes were selected such that statistical power >0.8 given our standard deviation and significance.

## Results

### Indole inhibits motility

**Fig. 1.**
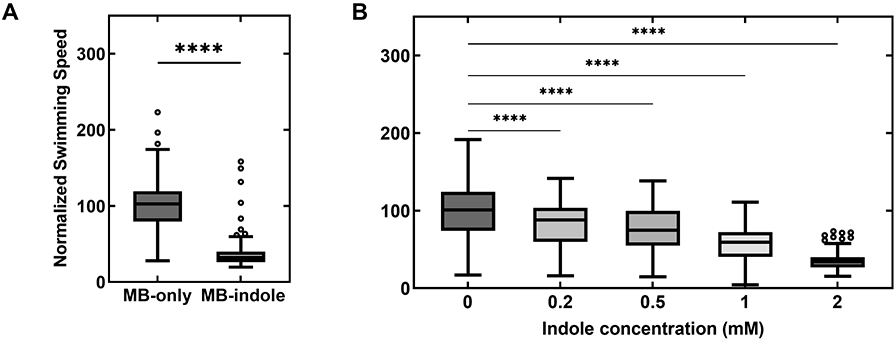
(A) The swimming speed of wild-type cells is indicated in MB with and without 2 mM indole. The speeds were normalized by the mean speed in MB-only (25.9 ± 8.6 μm/s). The horizontal line inside the box represents the median and the enclosing borders represent 25th and 75th percentiles. The lengths of the whiskers are based on Tukey’s criterion—1.5 times the interquartile range. Outliers are represented by open circles. (B) The normalized swimming speeds in a Δ*cheY* strain are plotted against increasing concentrations of indole. Differences in the means were statistically significant (*P*-value <0.0001), except between the data-points at 0.2 and 0.5 mM indole. Each mean value in (A) and (B) represents an average of *n* >100 swimming cells.

We measured indole’s effect on motility in wild-type *E. coli* by monitoring the swimming speeds of single cells in MB containing 2 mM indole. The swimming speeds were quantified from recorded videos and normalized with the mean swimming speed in MB-only control experiments (see Methods). The mean speed decreased by 63.2 ± 21.4% in the presence of indole (Fig. 1A). As indole induces more frequent tumbling in cells (16), the swimming speeds fluctuated significantly. Hence, we performed motility measurements in a smooth-swimming strain that lacked *cheY,* without which the motors rotate exclusively CCW. However, even in these smooth swimmers, we observed a consistent decrease in speeds over the range 0–2 mM indole (Fig. 1B). The decrease in the swimming speeds was ~24.7 ± 29.6% when cells were treated with 0.5 mM indole.

### Indole less than 1 mM does not affect membrane potential

**Fig. 2.**
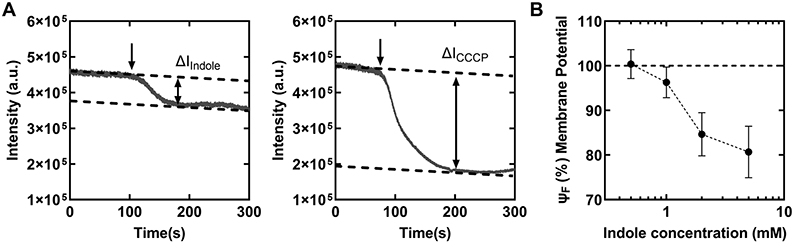
(A) *Left panel*: representative experiment shows the changes in the intensity of the fluorescence emissions from a population of cells (~500–1,000 cells) containing the ThT dye. The time of exposure of the cells to 5 mM indole is indicated by the downward arrow. The dotted lines indicate the steady-state pre- and post-stimulus values. The downward slope in signal was due to photobleaching. The relative signal reduction was calculated as the fractional decrease in the mean poststimulus value relative to the mean prestimulus value: ΔF_indole_ = (I_indole_^post^—I_indole_^pre^)/I_indole_^pre^ (see Methods). *Right panel:* the relative signal reduction upon exposure to 25 *μ*M CCCP is indicated: ΔF_CCCP_ = (I_CCCP_^post^—I_CCCP_^pre^)/I_CCCP_^pre^. **(B)** The change in the membrane potential, Ψ_F_ = (ΔF_CCCP_—ΔF_Indole_)/ΔF_CCCP_ × 100, is shown as a function of indole concentrations. The dotted line (Ψ_F_ = 100%) indicates the unperturbed membrane potential in 0 mM indole. The dotted curve is a guide to the eye.

We hypothesized that indole decreased the swimming speeds (Fig. 1) by inhibiting the membrane potential. To test this idea, we measured the effect of indole on the membrane potential in live populations of cells with the aid of a Nernstian fluorescent dye, ThT. ThT accumulates in the cell at high membrane potential and depletes as the potential dissipates (26,27). We first treated cells with 10 μM ThT, allowing adequate time (~30 min) for the cells to uptake the dye. Then, we stimulated the cells with indole solutions containing 10 μM ThT using perfusion chambers. Simultaneously, we excited cells with a low-intensity blue light and collected the emissions with the aid of a photomultiplier (see Methods). During stimulation, the dynamic changes in intensities were recorded as shown in Fig. 2A (left panel) to obtain relative signal reduction (Δ*F*_*indole*_) for each concentration of indole.

In separate experiments, we measured ΔF_CCCP_—the maximum possible relative reduction in the emission intensities (Fig. 2A, right panel)—by exposing the cells to 10 *μ*M ThT solutions containing 25 *μ*M carbonyl cyanide m-chlorophenyl hydrazine (CCCP), which is a powerful dissipater of the membrane potential (26, 28). We then normalized Δ*F*_*indole*_ at each indole concentration with ΔF_CCCP_ to obtain the relative changes in the membrane potential, Ψ_F_ (see Methods). A Ψ_F_ ~ 100% indicated no dissipation in the membrane potential, whereas Ψ_F_ ~ 0% indicated complete dissipation (Fig. 2B). As evident in Fig. 2B, 0.5 mM indole did not measurably impact the Ψ_F_. In total, 1 mM indole decreased the Ψ_F_ only by ~3.7 ± 3.2%. The maximum change in Ψ_F_ was observed between 1 and 2 mM indole and the changes appeared to plateau between 2 and 5 mM (~84.6 ± 4.8 and 80.7 ± 5.8%). Our observation that the Ψ_F_ remained unaffected in the presence of 0.5 mM indole suggests that the 24.7 ± 29.6% decrease in swimming speeds recorded at this indole concentration (Fig. 1B) was unlikely due to changes in the membrane potential.

### Indole induces CW rotation in motors in the absence of CheY

**Fig. 3.**
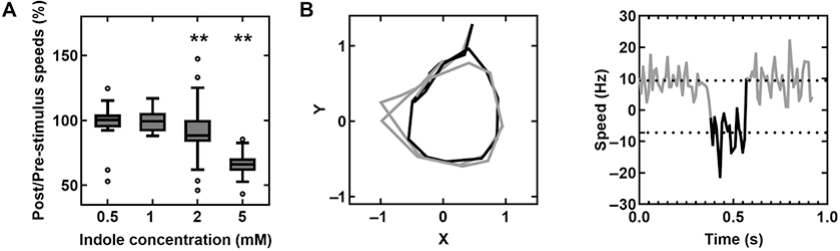
(A) The ratios of post- to pre-stimulus rotational speeds of CCW-only motors attached to 2 μm latex beads are indicated at varying concentrations of indole. The number of motors corresponding to the different treatments were: *n* = 17 (0.5 mM), 12 (1 mM), 45 (2 mM), and 25 motors (5 mM indole). Pairwise differences between pre- and post-stimulus values were significant only for motors treated with 2 and 5 mM indole (*P* <0.05). (B) *Left panel:* plot shows the x–y trajectory of a representative motor in the Δ*cheY* strain when it switched in the presence of 5 mM indole. The black data represent CW rotation; gray data indicate CCW rotation. *Right panel:* the corresponding speed trace for the same motor is shown as a function of time. The positive (negative) values indicate CCW (CW) speeds. The dotted lines indicate means speeds (9.4 ± 3.9 and −7.2 ± 7.6 *μ*m/s).

Our previous work indicated that even 2 mM indole did not affect cytosolic pH levels(16). As low concentrations of indole (<1mM) inhibited motility without significantly altering the membrane potential, we hypothesized that indole enters into the cytoplasm and influences motor behavior directly rather than by modulating PMF. To test this notion, we tracked the rotation of 2 μm latex beads stuck to short flagellar stubs in a Δ*cheY* strain in the presence of indole. Bead rotation was analyzed in recorded videos with particle-tracking algorithms to quantify the rotational speeds and the direction of rotation (see Methods). Below 1 mM indole, there was no significant decrease in the rotation speeds (Fig. 3A) even though the swimming speeds decreased significantly (Fig. 1B). The insensitivity of the speeds to indole at such low indole levels was consistent with the lack of changes in Ψ_F_ (Fig. 2B). As indole does not modulate the cytosolic pH (up to 2 mM indole, (16)), this suggested that indole did not inhibit motility by dissipating the PMF at lower concentrations. These observations also suggest that indole’s effects on motor speeds depend on the viscous load on the flagellar motor.

Above 1 mM indole, we observed ~8.8 ± 17.5% and 33.8 ± 9.8% decrease in the rotation speeds at 2- and 5-mM indole, respectively (Fig. 3A), consistent with the dramatic decreases in the Ψ_F_ at these indole concentrations (Fig. 2B). Interestingly, when we scanned motor traces visually, we observed occasional CW rotation in the motors of the Δ*cheY* strain. Several motors switched their direction of rotation in the presence of indole, sometimes undergoing 1 or 2 full CW rotations (see Fig. 3B and movie 1). Based on these and earlier findings, we propose that indole can partially complement CheY’s function. These observations are consistent with our previous hypothesis that indole modulates the free energies of the flagellar switch proteins to induce CW conformation in the flagellar switch (16).

### Indole modulates cooperative interactions within the switch complex to inhibit motility

The switch complex includes a ring of ~34 FliG subunits (29). The conformation of the FliG ring controls the direction of rotation. Binding of CheY-P to two additional complexes in the switch, FliM and FliN (30, 31), promotes CW conformation in the FliG ring: the direction changes from CCW to CW when all the FliG subunits cooperatively change their conformations from the CCW to the CW state (32, 33). The motor rotates at top speed for a given viscous load if all the FliG subunits simultaneously existed in the same conformation; when some subunits adopt the CW and others adopt the CCW conformation, the mixed conformations in the ring are predicted to cause slower rotation speeds (32, 33). This is because the multiple stator units that engage with different FliG subunits in different conformations generate opposing torques that tend to cancel each other. Based on our finding that indole can promote CW rotation even in the absence of CheY-P (Fig. 3B), we hypothesized that indole promotes mixed conformations in the FliG ring by increasing the probability that some FliG subunits will adopt the CW conformation, which could explain the Ψ_F_-independent inhibition of motility (Fig. 1B).

**Fig. 4.**
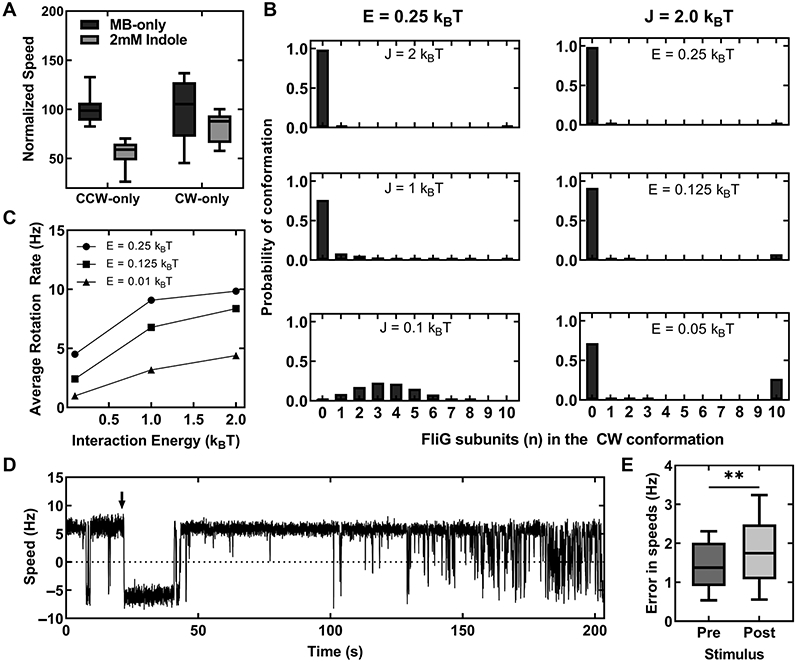
(A) Plot indicates a comparison between the mean rotational speeds 750-nm beads attached to the CCW-only and CW-only motors in MB solutions with and without 2 mM indole. The CCW-only motors were obtained in a Δ*cheY* strain and the CW-only motors were obtained in a Δ*cheY, fliG^CW^* strain. (B) The different panels indicate simulations of the effect of indole on the probabilities of observing *n* FliG subunits in the CW conformation (ordinate) in a ring consisting of *N* = 10 FliG subunits. *Left column*: attenuation of interaction energy (*J*) by indole increase the probability of observing multiple FliG subunits in the CW and CCW conformations simultaneously. The free energy difference in the CW-CCW conformations was held constant at *E* = 0.25 *k*_*B*_*T. Right*: attenuation of the free energy difference (*E*) between the CW and CCW states of the motor by indole increases the probability of observing CW rotation in a CCW-rotating motor. The strength of the interaction energy between FliG–FliG subunits was held constant at *J* = 2 k_B_T, where *k*_*B*_ is the Boltzmann’s constant and *T* is the absolute temperature. (C) The rotation speed predicted from Eq. 2 decreases as the interaction energy (*J*) decreases, irrespective of the value of *E*. (D) The rotation speeds of a representative wild-type tethered cell are indicated. The down arrow indicates the time of exposure to 1 mM indole, which caused the motor to momentarily rotate CW. As the motor adapted, CCW rotation increased. The noise in the rotation speeds increased ~100 s following the exposure to indole. (E) The difference in the mean standard deviations in the rotational speeds before and after treatment with 1 mM indole was statistically significant (*n* = 13 motors, *P*-value <0.05). The poststimulus noise was ~21% higher.

To test this, we repeated the bead assays in a Δ*cheY* strain that carried a mutant *fliG^CW^* allele in place of the native chromosomal *fliG* allele (32). The mutant FliG^CW^ subunits remain locked in the CW conformation causing the motors to rotate CW-only (34). As all the subunits are already in the CW conformation, indole is unlikely to induce mixed conformations within the FliG ring. We tested a smaller load (0.75 *μ*m beads) that approximates the viscous load experienced by swimming cells as the Ψ_F_-independent effects of indole on motor rotation appear to be stronger in swimming cells compared to cells rotating 2 *μ*m beads. As shown in Fig. 4A, the CW-only motor speeds decreased by ~17.8 ± 15.2% in the presence of 2 mM indole, similar to the 15.4 ± 4.8% decrease seen in Ψ_F_ at 2 mM indole (Fig. 2B). This suggests that the speed decrease was almost entirely due indole’s effect on the Ψ_F_, consistent with our hypothesis that a CW-locked FliG ring is less sensitive to indole’s effects on FliG conformations. In comparison, the mean speed of the CCW-only motors decreased by 45.1 ± 14.2% (Fig. 4A), presumably through a combination of Ψ_F_-dependent and Ψ_F_-independent causes. We also tested an alternate hypothesis in which indole degrades the ability of the stator proteins to associate with the motor. However, we did not find any such evidence to support this alternate hypothesis (see Figure S1, Supplementary Material). We conclude, therefore, that the attenuation in swimming speeds at low indole concentrations likely occurs because the metabolite promotes mixed conformations in the FliG ring. Higher viscous loads on the motor and locking of the FliG conformation in the CW state mitigate these effects.

To understand how indole could complement CheY-P (partially) and also inhibit motility without affecting Ψ_F_, we modeled the metabolite’s effects on the stochastic conformational changes in the FliG ring. We employed the conformational spread model, which has been successfully used previously to predict the effects of protein–protein cooperative interactions on the probability of observing mixed conformations in the switch complex (32, 33). Based on these earlier works, we assumed that the FliG ring in a Δ*cheY* strain can be represented as a 1D Ising ring consisting of *N* spins; each spin corresponding to a FliG subunit. The CCW and CW conformations of each subunit are represented by the orientations *ρ*_*i*_ = 1 and *ρ*_*i*_ = −1. The Hamiltonian is given by

(1) $$H_{k}=-J\sum \begin{array}{c} N\\ 1 \end{array}\sigma_{i}\sigma_{i+1}-E\sum \begin{array}{c} N\\ 1 \end{array}\sigma_{i},$$

with the periodic condition *ρ*_*N*+1_ = *ρ*_1_.

The symbol *E* represents the difference in the free energy between the high-energy CW and the low-energy CCW conformation; *J* represents the interaction energy between neighboring FliG subunits. The partition function, $Z=\sum \begin{array}{c} N\\ k \end{array}e^{H_{k}}$, was used to calculate *P*_*n*_, which is the probability of observing *n* FliG subunits in the CW conformation in an otherwise CCW ring of N subunits (see Supplementary Text). At *J* = 2 k_B_T and *E* = 0.5 k_B_T, which describe the flagellar switch energetics reasonably well (32), the FliG ring remained exclusively in the CCW conformation (Fig. 4B). However, assuming that indole decreases *E* correctly predicts an increase in the probability of CW rotation despite the absence of CheY, consistent with our observations in Fig. 3B. This lends support to the notion that indole influences intraprotein interactions.

Mere decreases in *E* though did not promote mixed conformations. Instead, the probabilities of observing mixed conformations increased only when indole was assumed to decrease the interaction energy, *J* (Fig. 4B). We predicted the dependence of the speed of rotation on the probabilities of mixed conformations from

(2) $$V_{avg}=V\left(\sum \begin{array}{c} \frac{N}{2}\\ n=1 \end{array}P_{n}(1-\gamma_{n})-\sum \begin{array}{c} N\\ n=\frac{N}{2} \end{array}P_{n}\gamma_{n}\right),$$

where *γ*_*n*_ = *n/N* and V is the maximum rotational speed at a particular load (see Supplementary Text). Equation 2 calculates the rotation speeds from the difference in the probabilities of FliG subunits existing in the CCW versus the CW conformations—when the probability of the entire ring existing in the CCW (or the CW) conformation is 1, the motor rotates at a maximum speed, ∣*V*∣. As indicated in Fig. 4C, the expression predicts that the rotational speed of the motor will decrease independent of Ψ_F_ if indole inhibits cooperative FliG–FliG interactions. As a final test of our hypothesis of mixed conformations, we quantified the variance in the rotational speeds of the flagellar motors in the absence and the presence of indole. The causes for the variance (noise) in rotation speeds can be grouped into 2: group 1, which depend on factors other than the internal dynamics of the motor (e.g. flagellar-tethering geometry, camera noise, and tracking errors) and group 2, which depend entirely on the internal dynamics such as the FliG ring conformations and stator function (including PMF fluctuations). Whereas group 1 noise is unlikely to be affected by indole, group 2 noise is likely to increase in the presence of indole as indicated by the increased probability of mixed conformations (Fig. 4B).

To quantify the noise observed in experimental traces of motor rotation, we calculated the standard deviation of speeds collected over 100 s durations in tethered cells. A representative rotational trace of a wild-type cell is indicated in Fig. 4D. When exposed to 1 mM indole, the noise increased in about ~130–200 s. A comparison of the noise in the rotational traces before and after treatment with indole in several motors revealed a slight increase in the noise due to indole (Fig. 4E). Although it is challenging to precisely estimate the changes in the energetics due to indole, the qualitative agreement between the model predictions and the experiments suggests that indole promotes mixed conformations in the FliG ring by weakening the cooperative interactions between subunits in the switch complex. We conclude that indole likely inhibits motility by modulating the free energy differences and the nearest-neighbor interactions in the FliG ring.

### Mtr is not necessary for indole transport

**Fig. 5.**
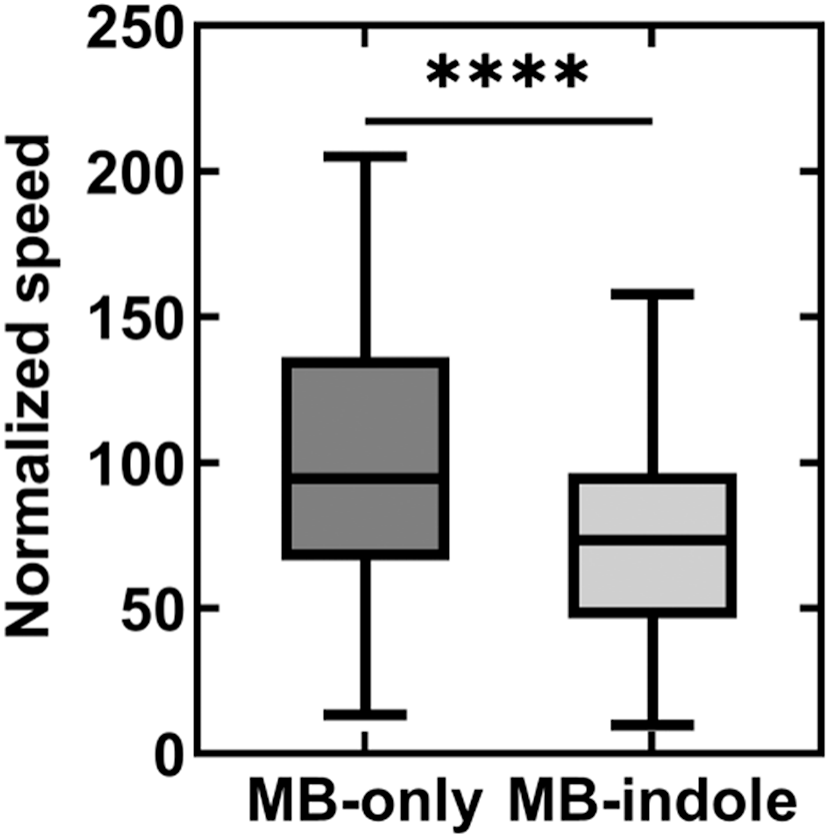
The mean swimming speed of an *mtr* mutant is indicated in the presence (*n* = 247 cells) and the absence of 0.5 mM indole (*n* = 345 cells). The speeds were normalized by the mean swimming speed in MB-only (motility buffer). The error bars indicate standard deviation. The swimming speeds pre and post indole treatment were statistically different (*P*-value <0.0001)

As indole modulates the interactions within the flagellar switch to inhibit motility, we used swimming speeds as a probe to investigate the role of the Mtr permease in indole’s entry into the cytoplasm. We quantified the effect of indole on the swimming speeds of a Δ*mtr* mutant that lacked CheY. The mean speed of the smooth-swimming cells was 28.4 ± 34.6% lower in the presence of 0.5 mM indole compared to MB-only solutions (Fig. 5). This matched well with the 24.7 ± 29.6% % decrease in swimming speeds in 0.5 mM indole when Mtr was present (Fig. 1B). This suggests that Mtr is not necessary for the cell to uptake exogeneous indole.

## Discussions

Our measurements indicate that indole inhibits motility in *E. coli* via several ways. At high concentrations (>1mM), indole decreased the PMF to dissipate the driving force, causing flagellar motors to rotate slower. Below 1 mM, however, indole did not significantly affect the PMF under our conditions (see the composition of our motility buffer in Methods). The decrease in swimming speeds at these low indole concentrations likely occurred because the metabolite interacted with the flagellar motor upon entering the cytoplasm. Although it is possible that indole affects stator–rotor interactions within the motor independent of any PMF-related effects, our experimental observations were more consistent with predictions from the conformational spread model, which assumed that indole disrupts cooperative interactions between FliG–FliG subunits, characterized by the interaction energy *J*. This promotes mixed conformations within the switch complex, which can cause individual stator units interacting with the different FliG subunits to deliver torque in opposite directions, ultimately decreasing the rotational speed. Further, we discovered that indole promoted CW rotation in the motor even in the absence of CheY. Presumably, indole is capable of modifying intraprotein interactions to decrease the free-energy difference, *E*, between the CW and the CCW conformations of individual FliG subunits. Considering that indole also activates different types of chemoreceptors in *E. coli* (16), it is possible that the metabolite is able to influence intraprotein and interprotein interactions in general, possibly by interacting nonspecifically with polar amino acid residues found in many proteins. If this is indeed the case, this general mechanism can explain indole’s wide-ranging effects on protein functions.

In *E. coli,* the increased probability of CW rotation corresponds to a repellent response. Our findings indicate that indole can induce a repellent response even in the absence of CheY. This provides a mechanism for the receptor-independent chemotactic response to the metabolite observed previously (16). We speculate that such rudimentary mechanisms to induce flagellar switching may have existed before the chemotaxis network was repurposed for sophisticated modulation of the switch behavior. It is possible that such motor-based response mechanism might be observable in other bacterial species. As receptor-based sensing can attract some cells to indole-rich niches, whereas motor-based responses primarily repel cells from those niches (16), it is possible that indole and other similar compounds in the exometabolome could present selective barriers for different bacteria: attracting some bacterial species while repelling others from metaboliterich niches. How the bacterial exometabolome selectively recruits bacterial communities to niches is a topic of ongoing study. As indole must enter the cell to interact with the flagellar motor, changes in motor function can indicate when the cell takes up the metabolite. We used this principle to determine that Mtr was not necessary for the uptake of exogenous indole in *E. coli.* Our single-cell motility results are consistent with passive diffusion of indole across the membrane.

As the flagellar rotational speeds are linearly proportional to the PMF (28), changes in the motor speeds can be used to quantitatively probe how chemicals influence the PMF (35). However, we have found that compounds such as indole can inhibit the rotational speeds of the flagellar motor even without dissipating the PMF. Further, indole’s PMF-independent effects on the motor speeds are also dependent on the viscous load on the motor: indole causes a greater decrease in rotation speeds at lower loads compared to higher loads. This can complicate the estimation of PMF changes from motor-based assays. According to our recent findings, high loads likely increase the free-energy difference (*E*), decreasing the propensity of a FliG subunit to adopt the CW conformation (36). Here, we propose that higher loads also increase the interaction energy, *J*, decreasing the likelihood of mixed FliG conformations. This could explain why indole’s effects on the motor were attenuated in our high load assays, until the metabolite concentrations increased adequately to dissipate the PMF. These observations provide a likely explanation for the discrepancies in the estimates of PMF dissipation by indole from low-load flagellar motor assays compared to fluorescence intensity measurements (13, 37, 38). For these reasons, we suggest that combining motor assays at high viscous loads with fluorescence-based detection approaches is a better strategy for reliably quantifying PMF changes. Interestingly, although both assays indicated that 2 mM indole decreased the PMF within minutes, the ATP levels did not decrease as our buffers contained lactate (see Figure S2A, Supplementary Material). Oxygen consumption however, did decrease in this duration (Figure S2B, Supplementary Material). It is possible that the cells use lactate in the buffer as a fermentative substrate to synthesize ATP and compensate for the decrease in the PMF (39-41). If so, the resultant byproducts (e.g. acetate) could further influence cellular physiology (42, 43). These effects of indole on protein interactions, cell energetics and metabolism are likely critical in its ability to impact diverse physiological functions.

## Supplementary Material

Movie 1 Movie 1: The movie shows a rotating 2 micron bead attached to a flagellar motor. The change in the color of the asterisk (*) from red to blue and vice versa corresponds to change in the direction of rotation.

Supplementary Text

## Figures and Tables

**Table 1.** List of strains.

Strain number	Genotype	References
PL77	Δ*cheY, fliC^st^*	(21)
PL141	Δ*cheY*	(22)
PL34	Δ*cheY, fliG*^CW^, *fliC*^*st*^	(23)
PL370	Δ*cheY* Δ*mtr* :: FRT-kan-FRT	This work

## Data Availability

Data is available in the manuscript and supplementary text. Parent strains are available from the Coli Genetic Stock Center. Minor derivatives of the parent strains (Table 1) are available upon email request to plele@tamu.edu.

## Significance Statement:

The exometabolome plays a key role in modulating the formation of bacterial communities. Indole, a major component of the exometabolome, likely influences colonization by regulating several aspects of bacterial physiology. However, the mechanisms are unknown. Here, we determined that indole inhibits motility by weakening cooperative protein interactions within the flagellum. We also discovered that indole is able to partially complement the function of the chemotaxis response regulator, CheY-P, by modulating protein conformations within the flagellar motor. Additionally, indole dissipated the membrane potential to inhibit motility. These findings offer major insights regarding a plausible general mechanism by which indole could modulate a variety of physiological functions.
