# Supplementary material for: Indole modulates cooperative protein–protein interactions in the flagellar motor
Source: PNAS Nexus. Author manuscript; Available in PMC 2022 Jun 17. (PMC9205328; doi:10.1093/pnasnexus/pgac035)
Supplement: Supplementary file 1 [file NIHMS1809097-supplement-Supplementary_Text.pdf]

Supplementary Text

Indole modulates cooperative protein-protein interactions in the flagellar motor

Rachit Gupta[1], Kathy Y. Rhee[1], Sarah D. Beagle[2], Ravi Chawla[3], Nicolas Perdomo[1], Steve W. Lockless[2] and Pushkar P. Lele[1] *

1. Artie McFerrin Department of Chemical Engineering, Texas A&M University, College Station, TX (USA) – 77843-3122.
2. Department of Biology, Texas A&M University, College Station, TX (USA) 77843-3258
3. Department of Integrative Structural and Computational Biology, Scripps Research, La Jolla, CA 92037.

*** Correspondence:**

Dr. Pushkar P. Lele

plele@tamu.edu

Keywords: Exometabolome, PMF, chemotaxis, switching, chemoreceptors

S1. Alternate model for indole action

The flagellar motor is rotated by several stator units. The speed of motor rotation increases as the number of bound stator units increases [1]. We reasoned that indole might weaken the association of the stator units with the motor to decrease its rotation speed. Hence, we overexpressed the stator proteins in the cell to test if the inhibitory effects of indole on motility could be alleviated by compensating for the weakened stator binding. We expressed MotA-MotB from a pTrc99A-based plasmid in the *cheY* mutant. We observed that even at high expression levels (IPTG = 100 μM), motility did not recover (**Fig S1A, left panel**). Next, we analyzed the rotation speeds when 750 nm beads were stimulated with 2 mM indole. The speeds decreased on stimulation and smoothly increased when indole was removed. We did not see evidence for step-wise increments during the recovery in speeds (**Fig. S1A, right panel**) – such increments occur when stator units re-associate with the motor (Blair & Berg, 1988). Very rarely were stepwise decreases observed in a few motors but these were limited to ~ 1 step. Hence, we conclude that indole does not significantly affect stator association with the motor.

S2. Varying external pH does not alleviate indole's effects on motility

We tested another possibility for the inhibition of motility by indole; we hypothesized that indole interfered with the flux of protons without affecting the membrane potential at low concentrations through some unknown mechanism. We decreased the external pH to increase the pH gradient across the membrane, thereby increasing the availability of protons. We adjusted the MB to pH 6 and measured motility over varying concentrations of indole. We observed a similar decrease in the swimming speeds as we had observed in the case of pH 7 (**Fig S1B**), consistent with the notion that indole does not act by modulating the proton flux especially at lower concentrations.

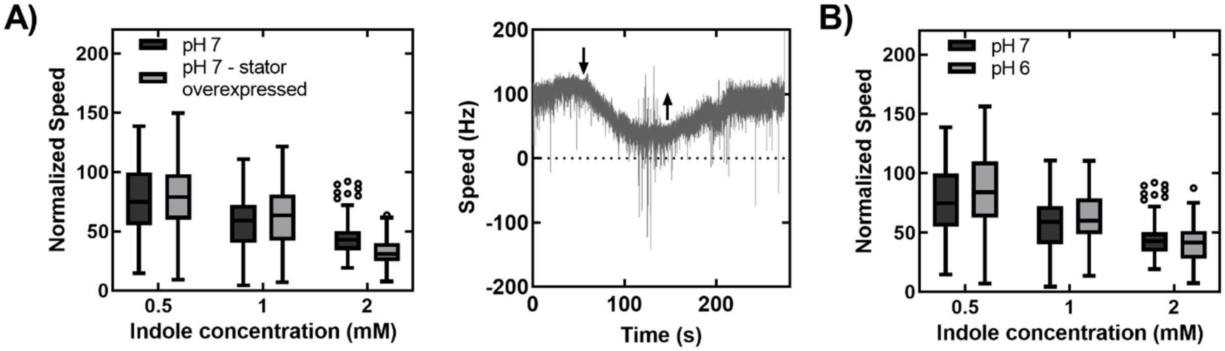

Figure S1: A) ***Left Panel:*** Effect of stator overexpression on swimming speeds in the presence of indole. Each data point was averaged over n > 100 swimming cells. Swimming speeds were normalized by the mean speed in MB-only. ***Right Panel*****:** The plot shows the speed of rotation of a single representative motor adhered to a 750 nM bead in a CCW-only strain. The downward arrow indicates time of exposure to 2mM indole and the upward arrow indicates the removal of indole. A smooth drop in rotation speed followed by a smooth recovery is visible. Similar behavior was observed for n = 11 motors. **B)** Effect of a decrease in the external pH on the smooth-swimming speeds is indicated in the presence of indole. The swimming speeds were normalized with the mean speed in MB-only. Each data point was averaged over n > 100 cells.

S3. Simulations for indole's effects on switch energetics (Conformational Spread Model)

The FliG ring is made up of several FliG subunits. Each subunit can be thought to exist in one of the two major conformations – a high energy and a low energy conformation, with the difference in free energies given by E. When all the FliG subunits adopt the same conformation, the motor rotates at a top speed for a particular viscous load [1]. Binding of CheY-P to FliM and FliN decreases E to promote CW rotation. During the switch in the direction of rotation, all the FliG subunits undergo a concerted change from one to the other conformation. The concerted changes in conformations are stabilized by interactions between FliG-FliG subunits, quantified by J in this work. It is possible for a fraction of the FliG subunits to be in a different conformation than the rest for a short period of time (~ few milliseconds). These transient mixed conformations can give rise to momentary

pauses in the rotation of the motor as the stator units interacting with FliG in opposite conformations deliver opposing torques [2].

The probabilities of observing the FliG ring in multi-conformational states can be estimated from a 1D Ising model (equation 1). We assumed that indole affected *E* and *J*, as discussed in the main-text. To quantify the effect of varying *E* and *J* on these probabilities, we simulated a ring consisting of N subunits with a periodic boundary. We assumed that there was no CheY present in the cell. The first step in calculating the probability of observing *n* subunits out of N FliG subunits in the CW state is to calculate the partition function. To calculate the partition function for the ring, we simulated the 2^N configurations that the ring could exist in. This is a non-trivial problem. Hence, we performed this task computationally for N = 10 subunits – this number was chosen rather than N = 34 to make the problem computationally tractable.

We began by assuming that all N subunits adopted the CCW conformation by default and initialized the ring with $\sigma = 1$ value (CCW conformation) assigned to each subunit. Then, we generated a binomial distribution: $S_n = \binom{n}{i}\pi^n(1-\pi)^{N-n}$, where π = 0.5 and *n* = 0, 1, 2 and so on to determine the probability of selecting *n* FliG subunits to be in the CW conformation. Each time a value for *n* was selected, we would randomly pick those many subunits from the ring and assign them a value $\sigma = -1$ (CW conformation) to realize a single configuration. The binomial distribution was employed as there are many more configurations possible for n = 5 compared to n = 1; the former case is simulated many more times as $S_5 >> S_1$. To ensure that all possible configurations were simulated, we performed a total of 10^6 simulations, well in the excess of the total configurations in the system. Next, we calculated the energies for each simulated configuration from equation 1 (main-text) and the partition function was determined by summing up over all the energies of the system, $Z = \sum_n \sum_j e^{-H_{n,j}}$, where *j* refers to the iterations performed for the nth value. Finally, the probability of observing *n* subunits in the CW conformation in an otherwise CCW ring was calculated as: $P_n = \sum_j e^{-H_{n,j}}/Z$. Increasing the total simulations events from 1 x 10^6 to 2 x 10^6 did not appreciably change the values of P_n.

Having obtained P_n, we determined the speed of rotation from equation 2 (main-text): $V_{avg} = \sum_{n=1}^{\frac{N}{2}} P_n V(1-\gamma_n) - \sum_{n=\frac{N}{2}}^{N} P_n V \gamma_n$. Here, $\gamma_n = n/N$ and V is the maximum rotational speed at a particular load. The expression assumes that the speed of rotation in the CCW (CW) direction is linearly proportional to the number of subunits in the CCW (CW) conformation. This is seen from the $V(1-\gamma_n)$ and $V\gamma_n$ terms on the right side of the equation. The contribution of each of the *n* cases to the overall speed in the CCW (CW) direction is weighted by the probability of observing each of those states. The difference accounts for the opposing forces developed by the stator units when interacting with FliG subunits in the opposite conformations.

S3. Indoles effect of ATP synthesis and Oxygen consumption rate

We tested if indole affected ATP synthesis considering that it dissipates the membrane potential at high concentrations and the F-ATPase is powered by the PMF [9]. Interestingly, our results indicate that even at 2 mM indole, ATP levels did not decrease. This is likely because the motility buffer contained lactate; when the buffer did not contain lactate, the ATP levels showed a clear decrease (**Fig S2A**). Our measurements further indicated that when lactate was present, ATP levels did not decrease even when cells were stimulated with the ionophore, CCCP (Carbonylcyanide-m-chlorophenylhydrazone). The ATP levels did decrease in the presence of CCCP when lactate was missing. These observations indicate that the cells rely on lactate to prevent ATP depletion in the presence of certain ionophores.

We observed that 2 mM indole decreased the consumption rate even in the presence of lactate (**Fig S2, right panel**). The effect of indole on the consumption of oxygen is similar to that of other protonophores [10, 11]. Thus, when stimulated by an ionophore, the cell likely uses a mechanism that does not involve aerobic respiration to maintain ATP levels in the presence of lactate. The exact mechanism is unknown.

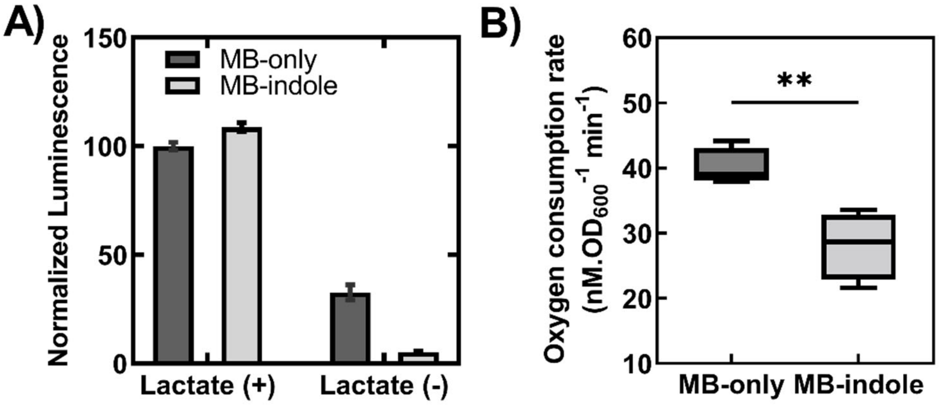

Figure S2: A) Normalized luminescence levels plotted on the y-axis against the media condition in the presence and absence of lactate in MB is indicated (refer Main text – Methods). Normalized Luminescence was obtained by dividing Luminescence by average luminescence measured for untreated cells multiplied by 100. In the presence of lactate, the luminescence levels appeared to slightly increase despite the reduction in membrane potential in the presence of 2 mM indole. **B)** The oxygen consumption rate (refer Methods from Main Text) is plotted on y axis against the media conditions (MB-only or MB-2mM indole) on x-axis. 2mM indole reduced the oxygen consumption by ~30%.

Supplementary Movie 1

The movie shows a rotating 2 micron bead attached to a flagellar motor. The change in the color of the asterisk (*) from red to blue and vice versa indicates the change in the direction of rotation.

References

1. Duke, T.A., Le Novère, N., and Bray, D. (2001). Conformational spread in a ring of proteins: a stochastic approach to allostery. J. Mol. Biol. ***308***, 541-553.

2. Bai, F., Branch, R.W., Nicolau, D.V., Pilizota, T., Steel, B.C., Maini, P.K., and Berry, R.M. (2010). Conformational Spread as a Mechanism for Cooperativity in the Bacterial Flagellar Switch. Science *327*, 685.

3. Ryu, W.S., Berry, R.M., and Berg, H.C. (2000). Torque-generating units of the flagellar motor of Escherichia coli have a high duty ratio. Nature *403*, 444-447.

4. Minamino, T., Imae, Y., Oosawa, F., Kobayashi, Y., and Oosawa, K. (2003). Effect of intracellular pH on rotational speed of bacterial flagellar motors. Journal of bacteriology *185*, 1190-1194.

5. Nakamura, S., Kami-ike, N., Yokota, J.-i.P., Kudo, S., Minamino, T., and Namba, K. (2009). Effect of Intracellular pH on the Torque–Speed Relationship of Bacterial Proton-Driven Flagellar Motor. Journal of Molecular Biology *386*, 332-338.

6. Yuan, J., and Berg, H.C. (2010). Thermal and solvent-isotope effects on the flagellar rotary motor near zero load. Biophysical journal *98*, 2121-2126.

7. Nord, A.L., Sowa, Y., Steel, B.C., Lo, C.-J., and Berry, R.M. (2017). Speed of the bacterial flagellar motor near zero load depends on the number of stator units. Proceedings of the National Academy of Sciences *114*, 11603.

8. Lo, C.-J., Leake, M.C., Pilizota, T., and Berry, R.M. (2007). Nonequivalence of Membrane Voltage and Ion-Gradient as Driving Forces for the Bacterial Flagellar Motor at Low Load. Biophysical Journal *93*, 294-302.

9. Mitchell, P. (1961). Coupling of Phosphorylation to Electron and Hydrogen Transfer by a Chemi-Osmotic type of Mechanism. Nature *191*, 144-148.

10. Goto, F., and Anraku, Y. (1974). Transport of sugars and amino acids in bacteria. IX. Studies on the active transport reaction in sodium azide- and 2,4-dinitrophenol-sensitive mutants of Escherichia coli. Journal of biochemistry *75*, 243-251.

11. Carneiro de Melo, A.M., Cook, G.M., Miles, R.J., and Poole, R.K. (1996). Nisin stimulates oxygen consumption by Staphylococcus aureus and Escherichia coli. Appl Environ Microbiol *62*, 1831-1834.
